# Hepatoprotective Effects of* Antrodia cinnamomea*: The Modulation of Oxidative Stress Signaling in a Mouse Model of Alcohol-Induced Acute Liver Injury

**DOI:** 10.1155/2017/7841823

**Published:** 2017-02-27

**Authors:** Yange Liu, Juan Wang, Lanzhou Li, Wenji Hu, Yidi Qu, Yipei Ding, Lina Meng, Lirong Teng, Di Wang

**Affiliations:** ^1^School of Life Sciences, Jilin University, Changchun 130012, China; ^2^Zhuhai College of Jilin University, Jilin University, Zhuhai 519000, China

## Abstract

In the present study, the components of* A. cinnamomea* (AC) mycelia were systematically analyzed. Subsequently, its hepatoprotective effects and the underlying mechanisms were explored using a mouse model of acute alcohol-induced liver injury. AC contained 25 types of fatty acid, 16 types of amino acid, 3 types of nucleotide, and 8 types of mineral. The hepatoprotective effects were observed after 2 weeks of AC treatment at doses of 75 mg/kg, 225 mg/kg, and 675 mg/kg in the mouse model. These effects were indicated by the changes in the levels of aspartate aminotransferase, alanine aminotransferase, several oxidation-related factors, and inflammatory cytokines in serum and/or liver samples. AC reduced the incidence rate of necrosis, inflammatory infiltration, fatty droplets formation, and cell apoptosis in liver detecting via histological and TUNEL assay. In addition, AC reduced the expression of cleaved caspase-3, -8, and -9 and the levels of phosphor-protein kinase B (Akt) and phosphor-nuclear factor-*κ*B (NF-*κ*B) in the liver samples. Collectively, AC-mediated hepatoprotective effects in a mouse model of acute alcohol-induced liver injury are the result of reduction in oxidative stress. This may be associated with Akt/NF-*κ*B signaling. These results provide valuable evidence to support the use of* A. cinnamomea* as a functional food and/or medicine.

## 1. Introduction

Alcohol metabolism and the associated oxidative stress and proinflammatory milieu in the liver can lead to hepatocellular injury [1, 2]. Alcoholic liver disease (ALD) develops in approximately 20% of alcoholics [1]. It is primarily caused by the byproducts of alcohol metabolism that promote the development of steatosis, which can lead to steatohepatitis, fibrosis, cirrhosis, and/or hepatocellular carcinoma [3]. ALD is a major health problem in the United States, accounting for 15% of the total healthcare costs, and it is associated with a mortality rate of 20% [4].

Under physiological conditions, reactive oxygen species (ROS) are efficiently eliminated by antioxidant defense systems, which involve enzymes that detoxify oxygen free radicals, such as superoxide dismutase (SOD) and glutathione peroxidase (GSH-Px) [5]. However, under pathological conditions, the overproduction of ROS induces apoptosis by activating proteins in the cysteine-dependent aspartate-directed protease (caspase) family and other signaling molecules such as nuclear factor-*κ*B (NF-*κ*B) and tumor necrosis factor-*α* (TNF-*α*) [6]. Interestingly, the overexpression of anti-inflammatory cytokines further accelerates cell damage [7].

Along with ROS, nitric oxide (NO) is involved in a wide range of toxic oxidative reactions [8]; therefore, inhibiting the release of NO from macrophages is a potential method of controlling inflammation [9, 10]. It has been demonstrated that oxidative stress is involved in the pathogenesis of ALD, as ROS generation has been observed in alcohol-exposed cultured cells and in alcohol-exposed mouse embryos [11]. It has also been shown that curcumin reduces inflammation by inhibiting NF-*κ*B in alcohol-exposed rats [3].

The most common medications for ALD are classified into three categories: supplemental raw materials for liver cell metabolism [12], opioid receptor antagonists [12], and agents that manage and improve alcohol metabolism [13, 14]. However, drug dependence, vomiting, dermatitis, dizziness, and leukopenia have been observed in ALD patients after long-term treatment with these agents [15]. Thus, there are currently no highly satisfactory therapeutic options for ALD. Many herbs and fungi have biological effects in humans and so they have been used as functional foods and medicines for centuries. In more recent years, many of these natural products have been used as the basis for the development of new biopharmaceuticals [16]. For example, genistein ameliorates alcohol-induced liver injury by reducing oxidative stress [17].

Another example is* Antrodia cinnamomea*, which is a basidiomycete that is found throughout Taiwan. It has multiple bioactive effects and it has traditionally been used as a health food [18]. One of the most effective ways to produce* A. cinnamomea* fruit bodies is to carry out submerged fermentation with the fungus. In total, 70 compounds have been isolated from* A. cinnamomea*, including polysaccharides, diterpenes, triterpenoids, fatty acids, amino acids, and steroids [19].* A. cinnamomea* has been shown to have hepatoprotective activity (as a result of its inhibition of free radical generation) in a rat model of liver injury [20]. In addition, it has also been shown to demonstrate hepatoprotective activity in rats with carbon tetrachloride-induced hepatotoxicity [21]. Although previous research has suggested that* A. cinnamomea* may act as a hepatoprotective agent, studies on the effects of* A. cinnamomea* mycelia (AC) on alcohol-induced liver injury (and the underlying mechanisms) remain rare.

First, we systematically analyzed the components of AC obtained from submerged fermentation. Subsequently, its hepatoprotective effects and the underlying mechanisms (related to the modulation of oxidative stress signaling) were explored in a mouse model of acute alcohol-induced hepatotoxicity. Our data provide valuable evidence to support the use of* A. cinnamomea* as a functional food and/or medicine.

## 2. Material and Methods

### 2.1. AC Culture and Sample Preparation

*A. cinnamomea* (ATCC 200183) was cultured in a liquid medium comprising 20 g/L glucose, 10 g/L yeast extract powder, 10 g/L tryptone, 1 g/L KH_2_PO_4_, 0.5 g/L MgSO_4_, and 0.1 g/L vitamin B_1_ with an initial pH ranging from 5.5 to 6.5. The AC were then collected and lyophilized for later use.

### 2.2. Measurement of the AC Components

#### 2.2.1. Main Components

The quantities of the main AC components—total protein, total sugar, reducing sugar, crude fat, triterpenoids, flavonoids, mannitol, adenosine, and total ash—were measured. The quantities were assessed using the Kjeldahl method [22], 3,5-dinitrosalicylic acid colorimetric estimation [23], phenol-sulfuric acid determination [23], the petroleum benzine extraction method, vanillin-glacial acetic acid and perchloric acid colorimetric spectrophotometry [24], the periodate oxidation method [25], an erinitrit-aluminium trichloride assay [23], a high-performance liquid chromatography analysis [23], and the ashing method [26], respectively.

#### 2.2.2. Amino Acids

The AC was hydrolyzed using 6 mol/L HCl at 110°C for 22 h. After vacuum drying, the samples were dissolved in 1 mL of a buffer with a pH of 2.2. A quantitative analysis of the amino acids was carried out using an automatic amino acid analyzer (L-8900, Hitachi, Japan).

#### 2.2.3. Nucleotides

The components of the AC were extracted using double distilled water at 50°C for 3 h and they were then centrifuged at 3500 rpm for 10 min. The nucleotides were analyzed using high-performance liquid chromatography with a C18 column (4.6 mm × 250 mm; 880975-902, Agilent, USA) and a UV detector (LC-20AD, Shimadzu, Japan) at 30°C. The mobile phase consisted of 5% methanol and 95% (50 mM) NaH_2_PO_4_. Adenosine monophosphate and uridine monophosphate were detected at 254 nm, hypoxanthine nucleotide was detected at 250 nm, and guanosine monophosphate was detected at 280 nm [27].

#### 2.2.4. Minerals

The AC was pretreated with hydrogen nitrate at a temperature of 110°C and an atmospheric pressure of 30 atm for 30 min. Subsequently, the levels of minerals, potassium (K), sodium (Na), calcium (Ca), magnesium (Mg), iron (Fe), zinc (Zn), selenium (Se), manganese (Mn), chromium (Cr), copper (Cu), lead (Pb), mercury (Hg), arsenic (As), and cadmium (Cd), were detected by inductively coupled plasma optical emission spectrometry [28].

#### 2.2.5. Fatty Acids

The components of the AC were extracted using a ratio of ether : petroleum ether of 1 : 1 (V : V). They were then mixed with 0.5 M NaOH in a methanol solution at 60°C for 30 min. A 25% BF_3_ solution was added to the samples and then they were incubated at 60°C for another 20 min. The samples were then mixed with a saturated solution of NaCl and hexane and the levels of fatty acids were analyzed using a gas chromatography-mass spectrometer (QP2010, Shimadzu, Japan) [29].

### 2.3. Animals and Experimental Design

The experimental protocol was approved by the Institutional Animal Ethics Committee of Jilin University. Sixty Kunming mice (8 weeks old; 18–22 g) (SCXK (JI)-2015-0047) were kept in an environmentally controlled room (at a temperature of 23°C ± 1°C and a relative humidity of 50% ± 10%) with a 12 h light-dark cycle and free access to water and food (except at night during the 2-week treatment period). The mice were acclimatized for 7 days and then they were randomly separated into six groups (with 10 mice in each group). Three of these groups were control groups (an alcohol-only group, a no-alcohol group, and a positive control group) and the other three were treated with AC.

The process of model development and drug treatment was similar to previous studies with some modification [30–32]. During the treatment period, after overnight fasting, all the mice except for those in the no-alcohol control group were orally given 9.52 g/kg white wine (Beijing Shunxin Agricultural Co. Ltd, China) with an alcohol degree of 56° once a day at 9:00 A.M. Once a day at 4:00 P.M., the mice in the positive control group were orally treated with 63 mg/kg silymarin (Sil; Madaus AG, Germany), which is a putative hepatoprotective agent that is extracted from the seeds of* Silybum marianum*. The mice in the three AC-treated groups were orally given 75 mg/kg, 225 mg/kg, and 675 mg/kg of AC, respectively, once a day at 4:00 P.M. The mice in the alcohol-only control group were orally given an equal volume of physiological saline once a day at 4:00 P.M. and the mice in the no-alcohol control group were orally given an equal volume of physiological saline twice a day at 9:00 A.M. and 4:00 P.M. During the 2-week treatment period, the behavior and bodyweight of each mouse were monitored daily.

### 2.4. Collection of Serum and Liver Samples

After the last treatment, each mouse was fasted overnight and a sample of blood was taken from its caudal vein. Each mouse was then sacrificed using an injection of 200 mg/kg pentobarbital and a sample of liver tissue was immediately collected and stored at −80°C.

### 2.5. Biochemical Assays

The levels of alanine aminotransferase (ALT) and aspartate aminotransferase (AST) in the serum and the levels of SOD, GSH-Px, ROS, and NO in the livers were measured using commercial diagnostic kits purchased from the Nanjing Jiancheng Institute of Biotechnology Co. Ltd. (Nanjing, China) in accordance with the instruction manuals. In addition, the levels of TNF-*α* and interleukin-10 (IL-10) in the serum and livers were measured using enzyme-linked immunosorbent assay (ELISA) kits obtained from Shanghai Yuanye Bio-Technology Co. Ltd. (Shanghai, China).

### 2.6. Histological Evaluation and TUNEL Assay

Following as previous description [33], liver tissues were fixed in 4% paraformaldehyde in 0.1 M phosphate buffer, dehydrated in graded alcohol, and embedded in paraffin and 5 *μ*m sections were prepared. Sections were stained with hematoxylin and eosin (H&E) for histological evaluation. All stained slides were visualized using an IX73 inverted microscope (40x; Olympus, Japan)

Cell apoptosis in liver tissues was detected by the terminal deoxynucleotidyl transferase-mediated dUTP nick end-labeling (TUNEL) kit (Life Technologies, USA) following the manufacturer's protocol. The changes of green fluorescence were determined by a fluorescent microscope (20x; CCD camera, TE2000, Nikon, Japan).

### 2.7. Western Blotting

For each mouse, a portion of the liver sample was homogenized in a lysis buffer, 1% of which consisted of a protease inhibitor cocktail (Sigma-Aldrich, USA), 2% of which was phenylmethanesulfonyl fluoride (Sigma-Aldrich, USA), and 97% of which consisted of the components of a radio-immunoprecipitation assay (Sigma-Aldrich, USA). The total protein concentration was measured using a bicinchoninic acid protein assay kit (Merck Millipore, Germany).

Sodium dodecyl sulfate-polyacrylamide gel electrophoresis (SDS-PAGE) was used to separate the proteins in 40 *µ*g of each liver sample. The SDS-PAGE was carried out using 12% polyacrylamide gel slabs and minivertical electrophoresis equipment (Bio-Rad, USA) and the proteins were electrotransferred onto 0.45 *µ*m nitrocellulose membranes (Millipore, USA). The membranes were blocked using 5% bovine serum albumin (BSA)/tris-buffered saline (TBS) at room temperature for 4 h. The blocked membranes were incubated in a 1000–2000-fold diluted solution of primary antibodies against phosphor (P)-Akt (07-1398; Merck Millipore, Germany), total (T)-Akt (ab131443), P-NF-*κ*B (ab25901), T-NF-*κ*B (ab7970), cleaved caspase-3 (ab13847; Abcam, USA), cleaved caspase-8 (ab25901), cleaved caspase-9 (ab25758), and glyceraldehyde-3-phosphate dehydrogenase (GAPDH; sc-25778; Santa Cruz Biotechnology, USA) at 4°C overnight.

Each membrane was washed five times with TBS plus Tween 20 and 5% BSA and it was then incubated with a 1500-fold diluted horseradish peroxidase-conjugated goat anti-rabbit secondary antibody (sc-3836; Santa Cruz Biotechnology, USA) at 4°C for 4 h. The proteins were visualized using a gel imaging system (UVP, USA). The intensity of each band was quantified using densitometric scanning with ImageJ software (National Institutes of Health, USA).

### 2.8. Statistical Analysis

The data were analyzed using SPSS 16.0 software (IBM corporation, USA). The results were presented as means ± standard errors of the mean (SEMs) and the statistical significance of each difference was determined using a one-way analysis of variance (ANOVA) followed by Dunn's test. *P* values of < 0.05 were considered to indicate statistically significant differences.

## 3. Results

### 3.1. Composition of AC

Of the constituents of AC, 11.7% was total sugar, 2.2% was reducing sugar, 8.05% was triterpenoids, 0.35% was flavonoids, 5.4% was mannitol, 30.01% was crude fat, 30.6% was total protein, and 0.16% was adenosine (Table 1).

A total of 35 types of fatty acid were detected but octanoic acid, tridecanoic acid, myristoleic acid, pentadecenoic acid, elaidic acid, linoleic acid, *α*-linolenic acid, docosadienoic acid, nervonic acid, and docosahexaenoic acid were not detected (Table 2). In addition, 16 types of amino acid were detected; the three most common ones were glutamic acid, arginine, and aspartic acid (Table 3). Furthermore, eight minerals and three nucleotides were observed in the following proportions: 77.2‱ K, 21.3‱ Na, 5.8‱ Ca, 16.8‱ Mg, 0.7‱ Fe, 0.6‱ Zn, 0.06‱ Mn, 0.1‱ Cr, 70000 mg/kg guanylic acid (GMP), 460.2 mg/kg uridylic acid (UMP), and 1142.1 mg/kg adenylic acid (AMP) (Table 4). Regarding the heavy metals, Pb, Hg, As, and Cd were not detected, and Cu was detected at a concentration of less than 20 parts per million, which is much lower than the official safety limits for humans (Table 4).

### 3.2. Hepatoprotective Effects of AC

During the 2-week treatment period, the bodyweight of the no-alcohol control mice increased by 44.1% (Table 5). In contrast, 2 weeks of alcohol consumption caused a 24.6% decrease in the bodyweight of the alcohol-only control mice, which began on the seventh day (*P* < 0.001; Table 5). Unlike the mice in the Sil-treated group, those in the AC-treated groups had bodyweight increases of up to 10% (beginning on the tenth day) compared to those in the alcohol-only control group (*P* < 0.05; Table 5).

**Figure 1:**
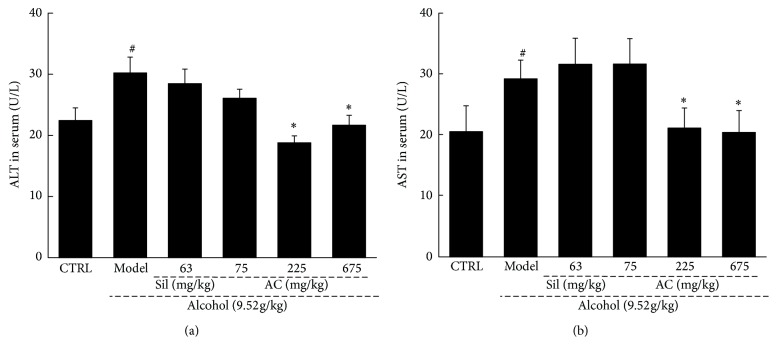
Two-week AC and Sil treatment reduced the levels of (a) ALT and (b) AST in the serum of mice with acute alcohol-induced liver injury. The data were analyzed using a one-way ANOVA and they are expressed as means SEMs (*n* = 10). ^#^*P* < 0.05 in a comparison with the no-alcohol control group; ^*∗*^*P* < 0.05 in a comparison with the alcohol-only control group. AC:* A. cinnamomea* mycelia; Sil: silymarin; ALT: alanine aminotransferase; AST: aspartate aminotransferase; ANOVA: analysis of variance; SEM: standard error of the mean.

The levels of ALT and AST activity in the serum, which are biochemical markers for assessing early-stage liver injuries, were examined to explore the effect of AC on acute alcohol-induced liver injury [34]. Compared to the no-alcohol control mice, the alcohol-only mice had strikingly increased levels of ALT and AST (*P* < 0.05; Figure 1), which were suppressed back to their normal levels by AC treatment at doses of 225 mg/kg and 675 mg/kg (*P* < 0.05; Figure 1).

### 3.3. Antioxidative Effects of AC

**Figure 2:**
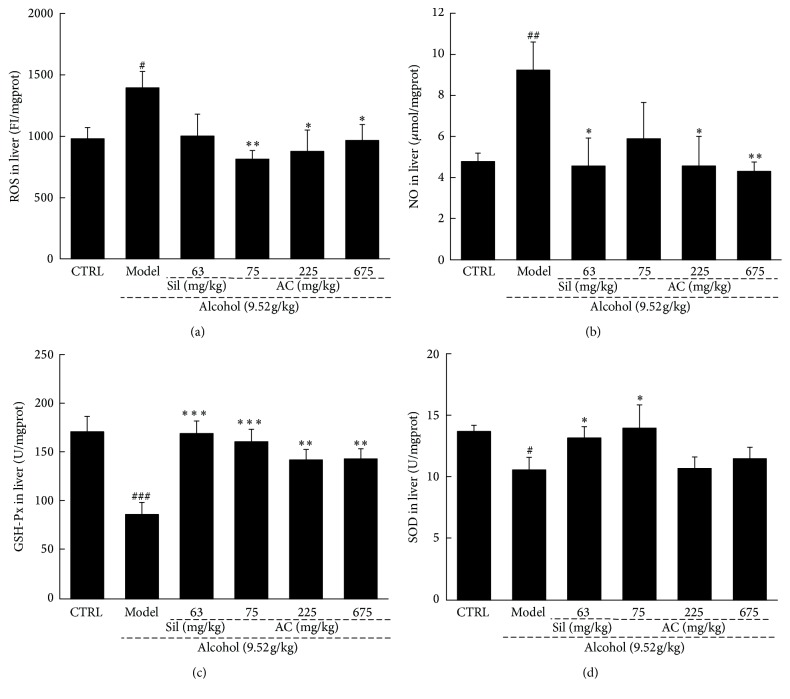
Two-week AC and Sil treatments affected the levels of (a) ROS, (b) NO, (c) GSH-Px, and (d) SOD in the livers of mice with acute alcohol-induced liver injury. The data were analyzed using a one-way ANOVA and they are expressed as means SEMs (*n* = 10). ^#^*P* < 0.05, ^##^*P* < 0.01, and ^###^*P* < 0.001 in a comparison with the no-alcohol control group; ^*∗*^*P* < 0.05, ^*∗∗*^*P* < 0.01, and ^*∗∗∗*^*P* < 0.001 in a comparison with the alcohol-only control group. AC:* A. cinnamomea* mycelia; Sil: silymarin; ANOVA: analysis of variance; GSH-Px: glutathione peroxidase; NO: nitric oxide; ROS: reactive oxygen species; SOD: superoxide dismutase; SEM: standard error of the mean.

ROS and NO levels can be used as markers of peroxidation and inflammation [9]. To assess the effects of AC on acute alcohol-induced hepatic oxidative stress, the levels of ROS and NO and the activities of SOD and GSH-Px were assessed. Extremely high ROS and NO levels and low SOD and GSH-Px activities were noted in the liver samples of mice with alcohol-induced hepatotoxicity (*P* < 0.05; Figure 2). Treatment with AC reversed these pathological changes and it even improved the levels of ROS, NO, GSH-Px activity, and SOD activity in the livers of the alcohol-only mice compared to those of the no-alcohol control mice (*P* < 0.05; Figure 2). In comparison, Sil only had beneficial effects on the levels of NO, GSH-Px activity, and SOD activity (not ROS) in the livers of alcohol-exposed mice (*P* < 0.05; Figure 2). These results demonstrated that AC may protect against alcohol-induced hepatic oxidative stress.

### 3.4. Effects of AC on Inflammatory Cytokines

To prove whether AC has anti-inflammatory effects, the levels of two important cytokines, IL-10 and TNF-*α*, in the serum and liver samples were assessed.

**Figure 3:**
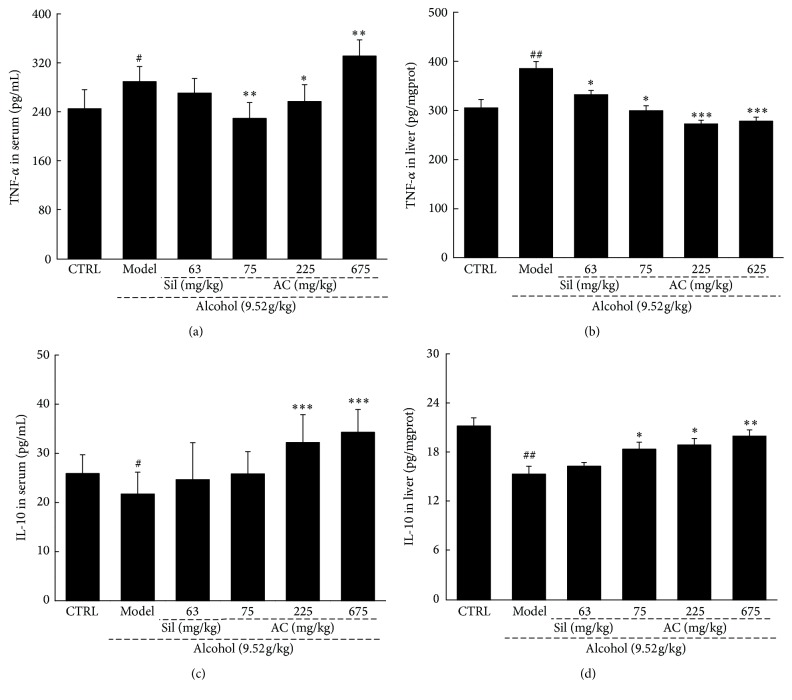
Two-week AC and Sil treatment (a and b) reduced the levels of TNF-*α* and (c and d) increased the levels of IL-10 in the serum and liver of mice with acute alcohol-induced liver injury. The data were analyzed using a one-way ANOVA and they are expressed as means SEMs (*n* = 10). ^#^*P* < 0.05 and ^##^*P* < 0.01 in a comparison with the no-alcohol control group; ^*∗*^*P* < 0.05, ^*∗∗*^*P* < 0.01, and ^*∗∗∗*^*P* < 0.001 in a comparison with the alcohol-only control group. AC:* A. cinnamomea* mycelia; Sil: silymarin; ANOVA: analysis of variance; SEM: standard error of the mean; IL-10: interleukin-10; TNF-*α*: tumor necrosis factor-*α*.

Acute alcohol exposure induced a dramatic increase in TNF-*α* levels in the serum and liver samples, and these increased levels were significantly suppressed by 2 weeks of treatment with AC (*P* < 0.05; Figures 3(a) and 3(b)). However, Sil only suppressed the increased levels of TNF-*α* in the liver samples and not in the serum samples (*P* < 0.05; Figure 3(b)).

Acute alcohol exposure significantly suppressed the levels of IL-10 in both the serum and liver samples (*P* < 0.05; Figures 3(c) and 3(d)). Treatment with AC increased the IL-10 levels in a dose-dependent manner in the serum and liver samples of mice with alcohol-induced liver damage compared with the alcohol-only control group, at doses ranging from 75 mg/kg to 675 mg/kg (*P* < 0.05; Figures 3(c) and 3(d)). However, treatment with Sil for 2 weeks failed to influence the IL-10 levels in the alcohol-exposed mice (Figures 3(c) and 3(d)).

### 3.5. Effect of AC on Histopathological Changes and Alcohol-Induced Apoptosis

**Figure 4:**
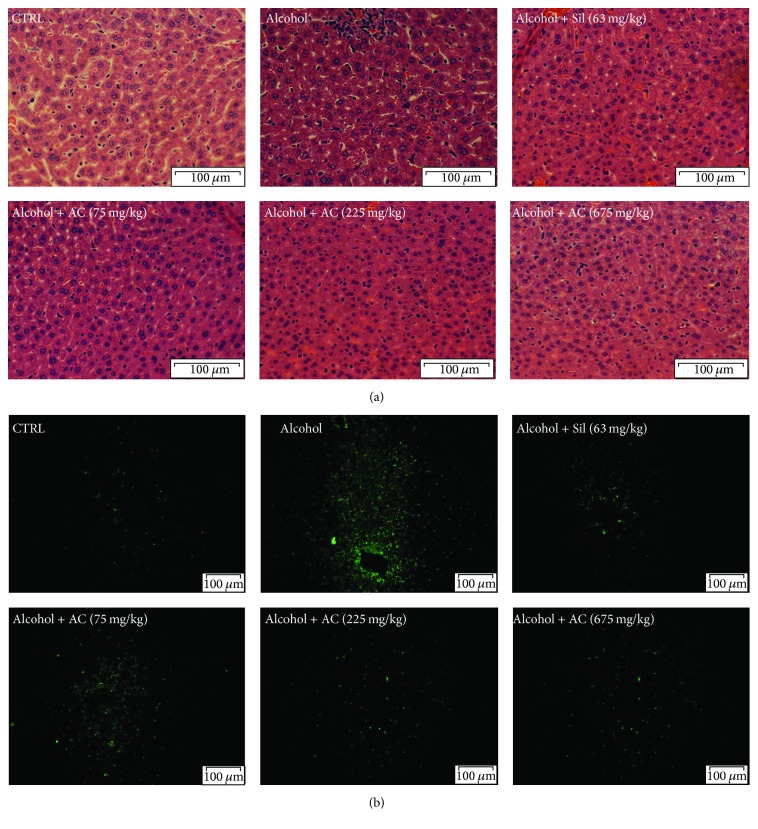
(a) Histopathological analysis in liver shown by H&E staining (scale bar: 100 *μ*m; magnification: 40x). (b) Apoptosis rate detection shown by TUNEL-positive cells with green fluorescence (scale bar: 100 *μ*m; magnification: 20x). AC:* A. cinnamomea* mycelia; Sil: silymarin; H&E: Hematoxylin and eosin; TUNEL: terminal deoxynucleotidyl transferase-mediated dUTP nick end-labeling.

H&E stain is the most fundamental and universal method for the histologic and pathologic examination [35]. Compared with control mice, alcohol-induced typical pathological characteristics in liver including necrosis, inflammatory infiltration of lymphocytes, and an increased number of fat droplets were noted (Figure 4(a)). In contrast, AC and Sil exhibited significant liver protection indicated by the reduced occurrence rate of necrosis, inflammatory infiltration, and fat droplets in liver of treated mice (Figure 4(a)).

TUNEL assay is performed to evaluate cell apoptosis condition in liver tissues. Large apoptotic cells with high intensity green fluorescence were noted in liver of alcohol-exposed mice (Figure 4(b)). Compared with model group, low cell apoptosis rate was observed in liver of AC- and Sil-treated mice (Figure 4(b)).

### 3.6. Effects of AC on the Activation of Akt and NF-*κ*B

**Figure 5:**
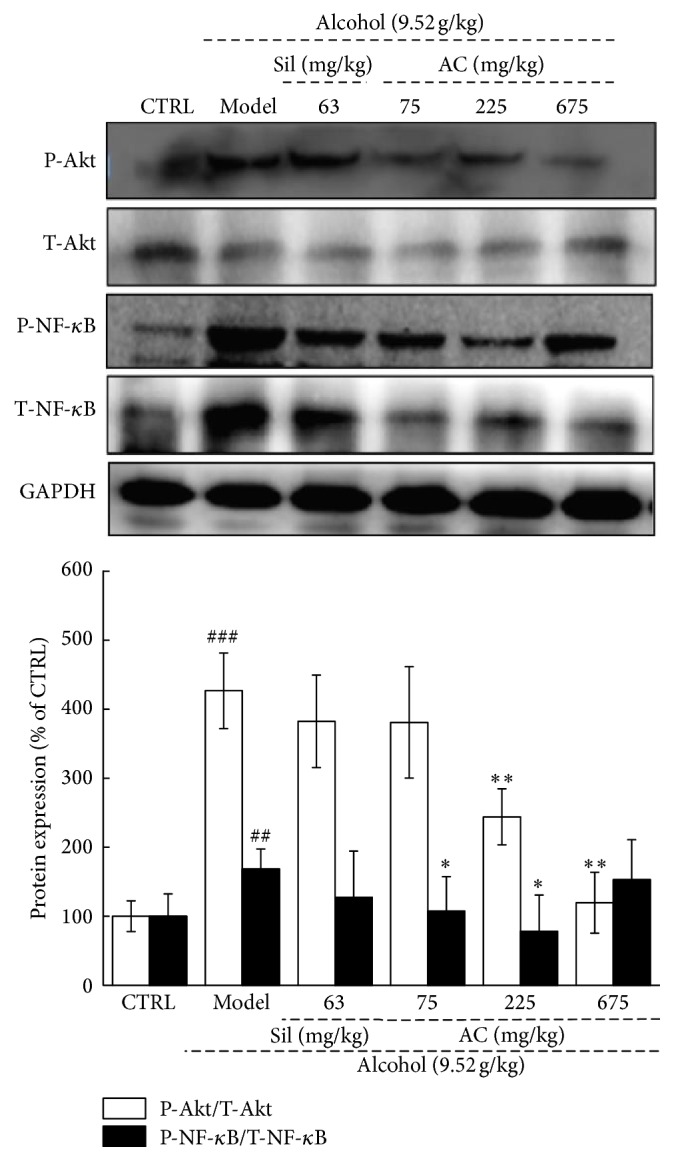
Two-week AC and Sil treatment suppressed the levels of phosphor-NF-*κ*B and phosphor-Akt in the livers of mice with acute alcohol-induced liver injury. The data on quantified protein expression were normalized to the levels of GAPDH. The data were analyzed using a one-way ANOVA and they are expressed as means SEMs (*n* = 10). ^##^*P* < 0.01 and ^###^*P* < 0.001 in a comparison with the no-alcohol control group; ^*∗*^*P* < 0.05 and ^*∗∗*^*P* < 0.01 in a comparison with the alcohol-only control group. AC:* A. cinnamomea* mycelia; Sil: silymarin; Akt: protein kinase B; ANOVA: analysis of variance; SEM: standard error of the mean; GAPDH: glyceraldehyde-3-phosphate dehydrogenase; NF-*κ*B: nuclear factor-*κ*B.

NF-*κ*B activation is involved in the positive regulation of TNF-*α* stimuli, and Akt is an important upstream activator in the inflammatory response involving NF-*κ*B and the generation of ROS [36]. The phosphorylation of both NF-*κ*B and Akt was remarkably increased in mice with alcohol-induced liver damage compared to the no-alcohol mice (*P* < 0.01; Figure 5). Unlike Sil, AC at doses from 75 mg/kg to 675 mg/kg resulted in a significant reduction in the expression of both phosphor-Akt and phosphor-NF-*κ*B in the livers of the alcohol-exposed mice (*P* < 0.05; Figure 5).

### 3.7. Effects of AC on the Expression of Caspases

**Figure 6:**
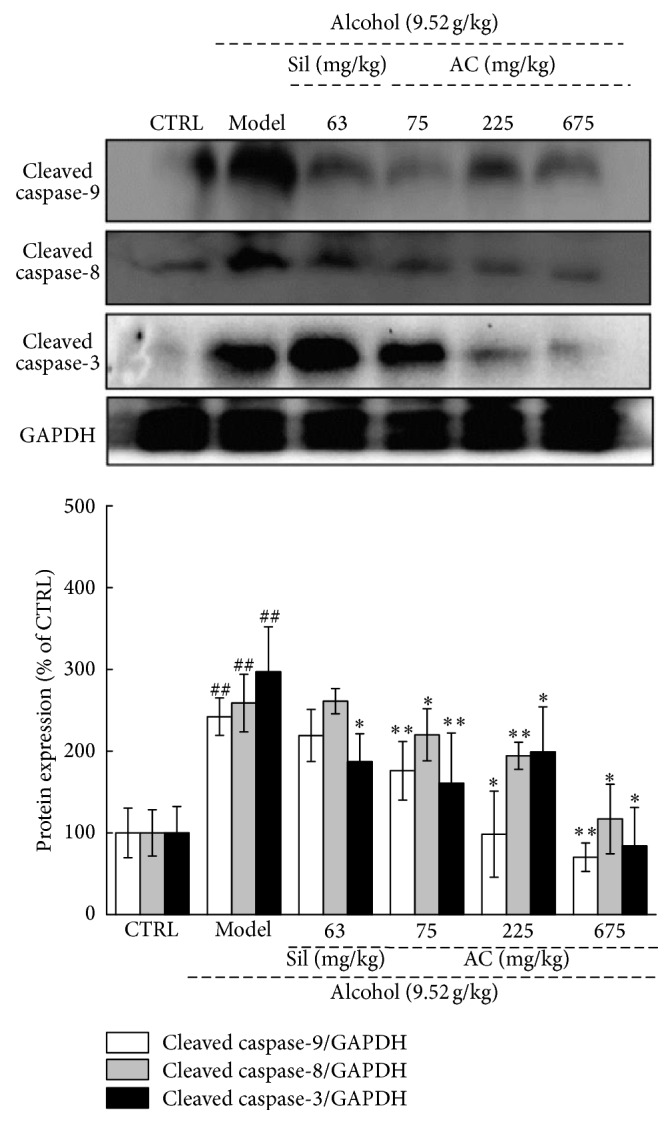
Two-week AC and Sil treatments suppressed the expression of cleaved caspase-3, -8, and -9 in the livers of mice with acute alcohol-induced liver injury. The data on quantified protein expression were normalized to the levels of GAPDH. The data were analyzed using a one-way ANOVA and they are expressed as means SEMs (*n* = 10). ^##^*P* < 0.01 in a comparison with the no-alcohol control group; ^*∗*^*P* < 0.05 and ^*∗∗*^*P* < 0.01 in a comparison with the alcohol-only control group. AC:* A. cinnamomea* mycelia; Sil: silymarin; ANOVA: analysis of variance; SEM: standard error of the mean; GAPDH: glyceraldehyde-3-phosphate dehydrogenase.

Oxidative stress is known to activate caspases which, in turn, cause cell damage [37]. Thus, to understand the mechanisms underlying the protective effect of AC against alcohol-induced oxidative stress in the liver, we assessed the expression of cleaved caspases-3, -8, and -9 in the liver samples. The expression of each of the three cleaved caspases was greatly increased in the livers of the alcohol-only mice compared to those of the no-alcohol mice (*P* < 0.01; Figure 6). Sil only reduced the expressions of cleaved caspases-3 (not cleaved caspases-8 and -9) in the alcohol-exposed mice (*P* < 0.05; Figure 6). Unlike Sil, treating with all doses of AC, there was significant depression in protein expression of cleaved caspase-3, -8, and -9 (*P* < 0.05; Figure 6). The effect of AC on caspases may be involved in its hepatoprotective effects against oxidative stress.

## 4. Discussion

We confirmed the hepatoprotective effects of AC in mice with acute alcohol-induced hepatotoxicity and clarified the underlying mechanisms of action, which are associated with the modulation of oxidative stress signaling. In contrast to other putative hepatoprotective agents (such as Sil), which are often extracted from plants,* A. cinnamomea* is an edible fungus that contains pharmacologically active ingredients [38]. In this study, 25 types of fatty acid, 16 types of amino acid, 3 types of nucleotide, and 8 types of mineral were detected in AC obtained from submerged fermentation.

Terpenoids, such as oleanolic acid, can increase the glutathione content of the liver, suppress lipid peroxidation, eliminate oxygen free radicals, and stimulate endogenous liver regeneration [39]. Flavonoids have protective effects against nonalcoholic steatohepatitis [40]. Our ongoing experiments focus on the hepatoprotective effects of purified terpenoids and flavonoids in alcohol-exposed mouse models similar to the one used in this study. The levels of the fatty acids oleic acid and translinoleic acid, which help to soften blood vessels and cannot be synthesized by the human body, were very high in the AC [29]. Furthermore, eicosapentaenoic acid, which was also detected in the AC, can reduce inflammatory immune responses and cure autoimmune diseases [41]. Together, these findings suggest that AC contains multiple active ingredients that can target various processes in the liver and can thereby prevent alcohol-induced liver damage. The presence of multiple active ingredients in AC may explain the fact that there was no dose-dependent response in our experiment. The absence of a dose-dependent response is a common feature of pharmaceutically active natural products [42].

The safety of* A. cinnamomea* has been verified by its use as a traditional functional food over several centuries. According to our data, many nonheavy metals were detected in AC. However, the heavy metals Pb, Hg, As, and Cd were not detected and Cu was present at a concentration of less than 20 parts per million, which further indicates the safety of AC. Encouragingly, in our study, the AC had no effect on the behavior of the mice (data not shown).

ALD is one of the most serious complications of heavy drinking. Liver serves as the main target organ of alcohol metabolism. Alcohol can cause several types of liver damage, including alcoholic hepatic steatosis, alcoholic hepatitis, fibrosis, and cirrhosis [43]. Severe liver damage occurred as a result of 2 weeks of alcohol administration, which was highlighted by large increases in the levels of AST and ALT activity, prooxidation enzymes, inflammatory cytokines, and caspases. These increased levels were all greatly decreased by AC. Additionally, the observed necrosis accumulation, inflammatory infiltration, fatty droplets formation, and cell apoptosis in liver of model mice provided clear evidence for alcohol-induced liver damage. The hepatoprotective effects of AC against alcohol-induced damage have been directly certified by reducing necrosis, inflammatory infiltration, fatty droplets formation, and apoptosis rate in liver.

Oxidative stress is a crucial causal factor of acute alcohol-induced liver injury. This is especially the case when the liver has lower levels of antioxidant protection to cope with the generation of ROS [44], which react with macromolecules including deoxyribonucleic acid (DNA), proteins, and the components of cellular membranes [45]. NO, another factor that is involved in oxidative stress, induces the formation of hydroxyl radicals, which are involved in alcohol-induced liver damage [46]. Targeting oxidative stress may prevent alcohol-induced liver injury.

AC not only suppressed the overproduction of ROS and NO, but also enhanced the activities of SOD and GSH-Px. The restoration of SOD and GSH-Px activity ameliorates alcohol-induced hepatotoxicity by reducing oxidative stress. A high level of SOD activity in liver cells diminishes oxygen free radicals and thereby reduces alcohol-induced hepatotoxicity [47]. Our data on a mouse model of alcohol-induced liver damage suggest that the antioxidative activities of AC are involved in its hepatoprotective effect.

Evidence has indicated that alcohol ingestion activates the innate immune system by changing the levels of inflammatory cytokines [48]. As a representative inflammatory cytokine with pleiotropic functions, TNF-*α* is closely associated with the progression of many inflammatory disorders [49]. Alcohol stimulates the production of TNF-*α*, and IL-10, a potent anti-inflammatory cytokine, can inhibit the production of TNF-*α* [50]. Mice injected with high doses of TNF can develop hepatocellular dysfunction with an elevation of serum levels of ALT and AST [51]. Previous studies have shown that oxidative stress leads to chronic inflammation [52]. However, during an inflammatory reaction, an increased oxygen uptake at the site of injury leads to a “respiratory burst” which, in turn, leads to an increased production and accumulation of ROS [53]. The generation of ROS is inhibited by TNF-*α*, which mediates a mild uncoupling of the mitochondrial respiratory chain in liver cells. The modulation of inflammatory cytokines in a mouse model of alcohol-induced liver damage by antioxidation factors therefore plays an important role in AC-mediated hepatoprotection.

The data revealed that AC strongly inhibits the phosphorylation of NF-*κ*B by inhibiting the phosphorylation of Akt, which controls NF-*κ*B activation via the activation of the I*κ*B kinase complex [54]. NF-*κ*B, a central transcription mediator, regulates the generation of inflammatory cytokines. The suppression of NF-*κ*B activation greatly decreases the levels of proinflammatory cytokines [55]. According to previous findings, ROS serves as a secondary messenger molecule between NF-*κ*B activation and TNF production [56]. TNF-*α* can influence cell activity by activating caspase-8 and -10 [57]. Moreover, ROS activates caspase-3, -8, and -9, which are cysteine-dependent proteases that mediate cell death [58]. Caspase-9 directly influences caspase-3 activation, usually by interacting with caspase-8 [58]. All the data suggest that the regulation of Akt/NF-*κ*B signaling (which is normally induced by oxidative stress) plays a central role in the hepatoprotective effect of AC against acute alcohol-induced liver damage.

In conclusion, AC, a nutritious natural product, has significant hepatoprotective effects on acute alcohol-induced hepatotoxicity in mice. It does this by regulating the levels of AST and ALT activity, oxidation-related enzymes, inflammatory cytokines, caspases; it may therefore influence Akt/NF-*κ*B signaling. Although the study may not provide sufficient evidence on the hepatoprotective effects of AC to support the use of AC as a medicine, it does provide evidence to support the use of AC as a functional natural product that can protect against ALD.

## Figures and Tables

**Table 1:** Main components of AC.

Compounds	Contents (%)
Total sugar	11.74
Reducing sugar	2.20
Triterpenoids	8.05
Flavonoids	0.35
Mannitol	5.37
Crude fat	30.01
Total ash	6.00
Total protein	30.60
Adenosine	0.16

AC: *A. cinnamomea* mycelia.

**Table 2:** The composition and percentage content of fatty acids.

Compounds	Contents (‱)
Octanoic acid (C8:0)	ND^①^
Capric acid (C10:0)	0.014
Undecanoic acid (C11:0)	0.052
Lauric acid (C12:0)	0.170
Tridecanoic acid (C13:0)	ND^①^
Myristic acid (C14:0)	6.541
Myristoleic acid (C14:1n5)	ND^①^
Pentadecanoic acid (C15:0)	1.616
Pentadecenoic acid (C15:1n5)	ND^①^
Hexadecanoic acid (C16:0)	66.599
Palmitoleic acid (C16:1n7)	0.300
Heptadecanoic acid (C17:0)	2.586
Heptadecenoic acid (C17:1n7)	1.704
Stearic acid (C18:0)	31.015
Elaidic acid (C18:1n9t)	ND^①^
Oleic acid (C18:1n9)	59.524
Translinoleic acid (C18:2n6t)	132.489
Linoleic acid (C18:2n6c)	ND^①^
Arachidic acid (C20:0)	0.535
*γ*-Linolenic acid (C18:3n6)	0.091
Paullinic acid (C20:1)	0.826
*α*-linolenic acid (C18:3n3)	ND^①^
Heneicosanoic acid (C21:0)	0.065
Eicosadienoic acid (C20:2)	0.642
Docosanoic acid (C22:0)	1.099
Dihomo-gamma-linolenic acid (C20:3n6)	0.018
Erucic acid (C22:1n9)	0.322
Eicosatrienoic acid (C20:3n3)	0.464
Arachidonic acid (C20:4n6)	0.098
Tricosanoic acid (C23:0)	0.091
Docosadienoic acid (C22:2n6)	ND^①^
Tetracosanoic acid (C24:0)	3.624
Eicosapentaenoic acid (C20:5n3)	0.303
Nervonic acid (C24:1n9)	ND^①^
Docosahexaenoic acid (C22:6n3)	ND^①^

^*①*^ND: not detected (the detection limit was 0.05 mg/kg).

**Table 3:** Percentage composition of amino acids in AC.

Compounds	Contents (%)
Aspartic acid (Asp)	2.86
L-Threonine (Thr)	1.53
Serine (Ser)	1.51
Glutamic acid (Glu)	4.44
Glycine (Gly)	2.25
Alanine (Ala)	2.34
Valine (Val)	1.66
DL-Methionine (Met)	1.01
Isoleucine (Iso)	1.23
Leucine (Leu)	2.42
Tyrosine (Tyr)	1.05
Phenylalanine (Phe)	1.38
Lysine (Lys)	2.3
Histidine (His)	0.89
Arginine (Arg)	3.19
Proline (Pro)	1.37

AC: *A. cinnamomea* mycelia.

**Table 4:** Percentage composition of nucleotides and minerals (including heavy metals) in AC.

Compounds	Contents
(‱)	
Kalium (K)	77.16
Natrium (Na)	21.29
Calcium (Ca)	5.82
Magnesium (Mg)	16.75
Ferrum (Fe)	0.67
Zinc (Zn)	0.62
Selenium (Se)	ND^②^
Manganese (Mn)	0.06
Chromium (Cr)	0.10

*(mg/kg)*	
Cuprum (Cu)	6.63
Lead (Pb)	ND^③^
Mercury (Hg)	ND^④^
Arsenic (As)	ND^⑤^
Cadmium (Cd)	ND^⑤^
Guanylic acid (GMP)	70000
Uridylic acid (UMP)	460.2
Adenylic acid (AMP)	1142.1

AC: *A. cinnamomea* mycelia.

^*②*^ND: not detected (the detection limit was 5 mg/kg).

^*③*^ND: not detected (the detection limit was 2 mg/kg).

^*④*^ND: not detected (the detection limit was 3 mg/kg).

^*⑤*^ND: not detected (the detection limit was 1 mg/kg).

**Table 5:** Effects of two-week AC treatment on the bodyweight of mice with acute alcohol-induced liver injury.

Days	CTRL	Model	Sil (mg/kg)	AC (mg/kg)		
			63	75	225	675
1st day	25.06 ± 0.51	25.87 ± 0.38	25.09 ± 0.39	25.90 ± 0.43	25.69 ± 0.29	26.30 ± 0.19
4th day	29.57 ± 0.53	27.55 ± 0.81	28.43 ± 0.61	29.77 ± 0.56	29.34 ± 0.52	28.31 ± 0.63
7th day	33.00 ± 0.58	27.77 ± 0.63^#^	29.04 ± 1.31	28.98 ± 0.70	29.85 ± 0.64^*∗*^	29.06 ± 0.59
10th day	33.90 ± 0.50	26.14 ± 0.94^##^	28.11 ± 1.00	29.15 ± 1.14^*∗*^	30.11 ± 0.78^*∗∗*^	29.00 ± 0.99^*∗*^
13th day	36.11 ± 0.36	27.22 ± 0.88^###^	29.53 ± 0.95	31.06 ± 1.02^*∗*^	29.94 ± 0.76^*∗*^	29.40 ± 0.91

The data were analyzed using a one-way ANOVA and they are expressed as means SEMs (*n* = 10). ^#^*P* < 0.05, ^##^*P* < 0.01, and ^###^*P* < 0.001 in a comparison with the no-alcohol control group; ^*∗*^*P* < 0.05 and ^*∗∗*^*P* < 0.01 in a comparison with the alcohol-only control group. AC: *A. cinnamomea* mycelia; Sil: silymarin.

## References

[1] Bakhautdin B., Das D., Mandal P. (2014). Protective role of HO-1 and carbon monoxide in ethanol-induced hepatocyte cell death and liver injury in mice. *Journal of Hepatology*.

[2] Chiu H.-W., Hua K.-F. (2016). Hepatoprotective effect of wheat-based solid-state fermented *antrodia cinnamomea* in carbon tetrachloride-induced liver injury in rat. *PLOS ONE*.

[3] Lee H.-I., McGregor R. A., Choi M.-S. (2013). Low doses of curcumin protect alcohol-induced liver damage by modulation of the alcohol metabolic pathway, CYP2E1 and AMPK. *Life Sciences*.

[4] Tang Y., Zhang L., Forsyth C. B., Shaikh M., Song S., Keshavarzian A. (2015). The role of miR-212 and iNOS in alcohol-induced intestinal barrier dysfunction and steatohepatitis. *Alcoholism: Clinical and Experimental Research*.

[5] Dai C., Li D., Gong L., Xiao X., Tang S. (2016). Curcumin ameliorates furazolidone-induced DNA damage and apoptosis in human hepatocyte L02 cells by inhibiting ROS production and mitochondrial pathway. *Molecules*.

[6] Ma K., Zhang C., Huang M.-Y., Guo Y.-X., Hu G.-Q. (2016). Crosstalk between Beclin-1-dependent autophagy and caspase-dependent apoptosis induced by tanshinone IIA in human osteosarcoma MG-63 cells. *Oncology Reports*.

[7] Wang T., Di G., Yang L. (2015). Saponins from Panax japonicus attenuate D-galactose-induced cognitive impairment through its anti-oxidative and anti-apoptotic effects in rats. *Journal of Pharmacy and Pharmacology*.

[8] Shibata K., Omahdi Z., Yamasaki S. (2016). Necroptosis DAMPens anti-tumor immunity. *Cell Death Discovery*.

[9] Cho M. L., Lee D.-J., Lee H.-S., Lee Y.-J., You S. G. (2013). LPS-induced NO inhibition and antioxidant activities of ethanol extracts and their solvent partitioned fractions from four brown seaweeds. *Ocean Science Journal*.

[10] Ganguli A., Choudhury D., Datta S., Bhattacharya S., Chakrabarti G. (2014). Inhibition of autophagy by chloroquine potentiates synergistically anti-cancer property of artemisinin by promoting ROS dependent apoptosis. *Biochimie*.

[11] Chen X., Liu J., Chen S.-Y. (2013). Sulforaphane protects against ethanol-induced oxidative stress and apoptosis in neural crest cells by the induction of Nrf2-mediated antioxidant response. *British Journal of Pharmacology*.

[12] Ozkol H., Bulut G., Balahoroglu R., Tuluce Y., Ozkol H. U. (2017). Protective Effects of selenium, N-acetylcysteine and vitamin E against acute ethanol intoxication in rats. *Biological Trace Element Research*.

[13] Higuera-de la Tijera F., Servín-Caamaño A. I., Serralde-Zúñiga A. E. (2015). Metadoxine improves the three- and six-month survival rates in patients with severe alcoholic hepatitis. *World Journal of Gastroenterology*.

[14] Tufton N., Hashim N., Sze C., Waterhouse M. (2015). A case of thyroid storm complicated by acute hepatitis due to propylthiouracil treatment. *Endocrinology, Diabetes & Metabolism Case Reports*.

[15] Triantafyllou K., Vlachogiannakos J., Ladas S. D. (2010). Gastrointestinal and liver side effects of drugs in elderly patients. *Best Practice and Research: Clinical Gastroenterology*.

[16] Camara-Clayette V., Lecluse Y., Schrader C. (2014). The NF-*κ*B pathway is rarely spontaneously activated in mantle cell lymphoma (MCL) cell lines and patient's samples. *European Journal of Cancer*.

[17] Zhao L., Wang Y., Liu J. (2016). Protective effects of genistein and puerarin against chronic alcohol-induced liver injury in mice via antioxidant, anti-inflammatory, and anti-apoptotic mechanisms. *Journal of Agricultural and Food Chemistry*.

[18] Chang C.-Y., Lee C.-L., Pan T.-M. (2006). Statistical optimization of medium components for the production of Antrodia cinnamomea AC0623 in submerged cultures. *Applied Microbiology and Biotechnology*.

[19] Qiao X., Song W., Wang Q. (2015). Comprehensive chemical analysis of triterpenoids and polysaccharides in the medicinal mushroom Antrodia cinnamomea. *RSC Advances*.

[20] Lu Z.-M., Tao W.-Y., Xu H.-Y., Ao Z.-H., Zhang X.-M., Xu Z.-H. (2011). Further studies on the hepatoprotective effect of Antrodia camphorata in submerged culture on ethanol-induced acute liver injury in rats. *Natural Product Research*.

[21] Song T.-Y., Yen G.-C. (2003). Protective effects of fermented filtrate from Antrodia camphorata in submerged culture against CCl4-induced hepatic toxicity in rats. *Journal of Agricultural and Food Chemistry*.

[22] Wang H., Pampati N., McCormick W. M., Bhattacharyya L. (2016). Protein nitrogen determination by kjeldahl digestion and ion chromatography. *Journal of Pharmaceutical Sciences*.

[23] Zhang N., Li Q., Wang J. (2014). Screening of Irpex lacteus mutant strains and optimizing fermentation conditions. *Journal of Food, Agriculture & Environment*.

[24] Ma T.-W., Lai Y., Yang F.-C. (2014). Enhanced production of triterpenoid in submerged cultures of Antrodia cinnamomea with the addition of citrus peel extract. *Bioprocess and Biosystems Engineering*.

[25] Khoigani S. R., Rajaei A., Goli S. A. (2016). Evaluation of antioxidant activity, total phenolics, total flavonoids and LC-MS/MS characterisation of phenolic constituents in Stachys lavandulifolia. *Natural Product Research*.

[26] Jurak E., Punt A. M., Arts W., Kabel M. A., Gruppen H. (2015). Fate of carbohydrates and lignin during composting and mycelium growth of Agaricus Bisporus on wheat straw based compost. *PLoS ONE*.

[27] Serra S., Deaglio S. (2016). HPLC-based assay to monitor extracellular nucleotide/nucleoside metabolism in human chronic lymphocytic leukemia cells. *Journal of Visualized Experiments*.

[28] Hurel C., Taneez M., Volpi Ghirardini A., Libralato G. (2017). Effects of mineral amendments on trace elements leaching from pre-treated marine sediment after simulated rainfall events. *Environmental Pollution*.

[29] Granata M. U., Bracco F., Gratani L. (2016). Fatty acid content profile and main constituents of *Corylus avellana* kernel in wild type and cultivars growing in Italy. *Natural Product Research*.

[30] Lim J. D., Lee S. R., Kim T. (2015). Fucoidan from fucus vesiculosus protects against alcohol-induced liver damage by modulating inflammatory mediators in mice and Hepg2 cells. *Marine Drugs*.

[31] Han Y., Xu Q., Hu J.-N., Han X.-Y., Li W., Zhao L.-C. (2015). Maltol, a food flavoring agent, attenuates acute alcohol-induced oxidative damage in mice. *Nutrients*.

[32] Li H.-H., Doiron K., Patterson A. D., Gonzalez F. J., Fornace A. J. (2013). Identification of serum insulin-like growth factor binding protein 1 as diagnostic biomarker for early-stage alcohol-induced liver disease. *Journal of Translational Medicine*.

[33] Relja B., Weber R., Maraslioglu M. (2015). Differential relevance of NF-*κ*B and JNK in the pathophysiology of hemorrhage/resususcitation-induced liver injury after chronic ethanol feeding. *PLoS ONE*.

[34] Okiyama W., Tanaka N., Nakajima T. (2009). Polyenephosphatidylcholine prevents alcoholic liver disease in PPAR*α*-null mice through attenuation of increases in oxidative stress. *Journal of Hepatology*.

[35] Pal S., Bhattacharjee A., Mukherjee S., Bhattacharya K., Mukherjee S., Khowala S. (2014). Effect of alocasia indica tuber extract on reducing hepatotoxicity and liver apoptosis in alcohol intoxicated rats. *BioMed Research International*.

[36] Liu J., Du L. (2015). PERK pathway is involved in oxygen-glucose-serum deprivation-induced NF-kB activation via ROS generation in spinal cord astrocytes. *Biochemical and Biophysical Research Communications*.

[37] Ishaq M., Khan M. A., Sharma K., Sharma G., Dutta R. K., Majumdar S. (2014). Gambogic acid induced oxidative stress dependent caspase activation regulates both apoptosis and autophagy by targeting various key molecules (NF-*κ*B, Beclin-1, p62 and NBR1) in human bladder cancer cells. *Biochimica et Biophysica Acta*.

[38] Kumar K. J. S., Chu F.-H., Hsieh H.-W. (2011). Antroquinonol from ethanolic extract of mycelium of *Antrodia cinnamomea* protects hepatic cells from ethanol-induced oxidative stress through Nrf-2 activation. *Journal of Ethnopharmacology*.

[39] He F., Bi H.-C., Xie Z.-Y. (2007). Rapid determination of six metabolites from multiple cytochrome P450 probe substrates in human liver microsome by liquid chromatography/mass spectrometry: application to high-throughput inhibition screening of terpenoids. *Rapid Communications in Mass Spectrometry*.

[40] Shin J. H., Jung J. H. (2016). Non-alcoholic fatty liver disease and flavonoids: current perspectives. *Clinics and Research in Hepatology and Gastroenterology*.

[41] Morin C., Blier P. U., Fortin S. (2015). Eicosapentaenoic acid and docosapentaenoic acid monoglycerides are more potent than docosahexaenoic acid monoglyceride to resolve inflammation in a rheumatoid arthritis model. *Arthritis Research and Therapy*.

[42] Ma L., Zhang S., Du M. (2015). Cordycepin from *Cordyceps militaris* prevents hyperglycemia in alloxan-induced diabetic mice. *Nutrition Research*.

[43] Cao Y.-W., Jiang Y., Zhang D.-Y. (2015). Protective effects of Penthorum chinense Pursh against chronic ethanol-induced liver injury in mice. *Journal of Ethnopharmacology*.

[44] Diaz-Aguirre V., Velez-Pardo C., Jimenez-Del-Rio M. (2016). Fructose sensitizes Jurkat cells oxidative stress-induced apoptosis via caspase-dependent and caspase-independent mechanisms. *Cell Biology International*.

[45] Li Y., Liu B., Yang F. (2016). Lobaplatin induces BGC-823 human gastric carcinoma cell apoptosis via ROS- mitochondrial apoptotic pathway and impairs cell migration and invasion. *Biomedicine and Pharmacotherapy*.

[46] Yamasaki H., Shimoji H., Ohshiro Y., Sakihama Y. (2001). Inhibitory effects of nitric oxide on oxidative phosphorylation in plant mitochondria. *Nitric Oxide*.

[47] Wu N., Shen H., Liu H., Wang Y., Bai Y., Han P. (2016). Acute blood glucose fluctuation enhances rat aorta endothelial cell apoptosis, oxidative stress and pro-inflammatory cytokine expression in vivo. *Cardiovascular Diabetology*.

[48] Zhao Z., Gong S., Wang S., Ma C. (2015). Effect and mechanism of evodiamine against ethanol-induced gastric ulcer in mice by suppressing Rho/NF-*κ*B pathway. *International Immunopharmacology*.

[49] Nepal S., Kim M. J., Subedi A. (2012). Globular adiponectin inhibits ethanol-induced apoptosis in HepG2 cells through heme oxygenase-1 induction. *Biochemical Pharmacology*.

[50] Denys A., Udalova I. A., Smith C. (2002). Evidence for a dual mechanism for IL-10 suppression of TNF-*α* production that does not involve inhibition of p38 mitogen-activated protein kinase or NF-*κ*B in primary human macrophages. *The Journal of Immunology*.

[51] Buffler M., Becker C., Windisch W., Schümann K. (2015). nflammation neither increases hepatic hepcidin nor affects intestinal ^59^Fe-absorption in two murine models of bowel inflammation, hemizygous TNF^ΔARE/+^ and homozygous IL-10^−/−^ mice. *Journal of Trace Elements in Medicine and Biology*.

[52] Cannon A. R., Morris N. L., Hammer A. M. (2016). Alcohol and inflammatory responses: Highlights of the 2015 Alcohol and Immunology Research Interest Group (AIRIG) meeting. *Alcohol*.

[53] Melichar B. (2016). Biomarkers, inflammation and cancer: where to go?. *Clinical Chemistry and Laboratory Medicine*.

[54] Shin S. Y., Kim C. G., Jung Y. J., Lim Y., Lee Y. H. (2016). The UPR inducer DPP23 inhibits the metastatic potential of MDA-MB-231 human breast cancer cells by targeting the Akt-IKK-NF-*κ*B–MMP-9 axis. *Scientific Reports*.

[55] Jiang T., Tian F., Zheng H. (2014). Nrf2 suppresses lupus nephritis through inhibition of oxidative injury and the NF-*κ*B-mediated inflammatory response. *Kidney International*.

[56] Averill-Bates D. A., Pallepati P. (2010). Activation of apoptosis by hydrogen peroxide through death receptor signaling is inhibited by mild heat preconditioning at 40°C. *Free Radical Biology and Medicine*.

[57] Kastl L., Sauer S., Beissbarth T., Becker M., Krammer P., Gülow K. (2014). TNF-a stimulation enhances ROS-dependent cell migration via NF-*κ*B activation in liver cells. *Free Radical Biology and Medicine*.

[58] Lv Q.-Y., Wan B., Guo L.-H., Zhao L., Yang Y. (2015). In vitro immune toxicity of polybrominated diphenyl ethers on murine peritoneal macrophages: apoptosis and immune cell dysfunction. *Chemosphere*.

